# Are Patient-Reported Outcome Measures for Anterior Cruciate Ligament Injuries Validated for Spanish Language and Culture?

**DOI:** 10.1177/23259671241256413

**Published:** 2024-08-09

**Authors:** Jeremy W. Siu, Edgar Garcia-Lopez, Nirav K. Pandya, Brian Feeley, Lauren M. Shapiro

**Affiliations:** † School of Medicine, University of California San Francisco, San Francisco, California, USA; ‡ Department of Orthopedic Surgery, University of California San Francisco, San Francisco, California, USA; Investigation performed at University of California, San Francisco, California, USA

**Keywords:** ACL, culture, language, patient-reported outcome measures, Spanish, validity

## Abstract

**Background:**

Patient-reported outcome measures (PROMs) have been adopted as a way to measure patient self-rated physical function and health status for patients with anterior cruciate ligament (ACL) injuries. Although multiple PROMs exist and have been translated into various languages, the cross-cultural adaptation and validity of these PROMs for Spanish-speaking patients is unknown.

**Purpose:**

To evaluate the adaptation quality and psychometric properties of Spanish-language adaptations of PROMs for patients with ACL injuries.

**Study Design:**

Scoping review; Level of evidence, 3.

**Methods:**

Under PRISMA (Preferred Reporting Items for Systematic Reviews and Meta-Analyses) guidelines, we reviewed published studies related to adaptation quality and psychometric properties of Spanish PROMs in patients with ACL injuries. The methodological quality of the included studies was assessed using the Guidelines for the Process of Cross-Cultural Adaptation of Self-Reported Measures, the Quality Criteria for Psychometric Properties of Health Status Questionnaires, and the Consensus-based Standards for the selection of health Measurement INstruments (COSMIN) checklist. The level of evidence for each PROM was determined based on the number of studies, methodological quality, consistency of results, and sample size.

**Results:**

The initial search strategy identified 5687 articles. After removal of duplicates, 1882 titles were screened, and 114 articles were assessed for eligibility. Six articles were selected for final review, comprising 4 PROMs: the Lysholm knee score, the Anterior Cruciate Ligament–Return to Sport After Injury (ACL-RSI), the Lower Extremity Functional Scale, and the Lower Limb Functional Index. Three studies followed all 6 processes for cross-cultural adaptation. None of the studies demonstrated all 14 domains required for cross-cultural validity (eg, description of translator expertise). The ACL-RSI achieved the highest level of evidence, with 3 of 9 domains demonstrating moderate evidence.

**Conclusion:**

This review identified 4 instruments that have been translated for Spanish-speaking patients with ACL injuries, none of which demonstrated appropriate adaptation or robust psychometric properties. The study highlights the need for improvement in PROMs for Spanish-speaking patients and the potential for mismeasurement and inappropriate application of PROM results in patients with ACL injuries.

Anterior cruciate ligament (ACL) injuries are among the most common orthopaedic injuries in the United States and around the world, with a global incidence estimated to be 29 to 39 per 100,000 persons.^16-18,26,27,44^ Patient-reported outcome measures (PROMs) are utilized alongside objective measures such as knee stability and range of motion to assess function, treatment outcomes, and quality of life after ACL injury. Previous studies have reported the Lysholm knee score and the International Documentation Committee (IKDC) Subjective Knee Form (SKF) as the most commonly used PROMs.^29^

The majority of PROMs have been developed in English-speaking populations, and validation studies often lack data related to the ethnic or cultural backgrounds of the study population.^14,19,28,30,48,55^ As such, the questions and responses on these PROMs may not apply to, or be valid for, patients of different ethnic or cultural backgrounds.^19,21^ For example, Paz et al^38^ demonstrated that English- and Spanish-speaking patients responded to 44% of items differently on linguistically translated PROMs, despite having similar levels of underlying physical function. This is particularly important for Spanish-speaking patients. Spanish is the second most widely spoken language globally; more than 500 million people speak Spanish as their first or second language, and 54 million people speak Spanish in the United States alone.^47^ Failing to utilize culturally appropriate PROMs can result in missing or inaccurate data due to misunderstood or culturally irrelevant items.^56^ This is reflective of a larger trend in which diverse patient populations are not included in orthopaedic research.^5^ Therefore, it has been recommended by governing bodies, including the US Food and Drug Administration (FDA), to use PROMs that have been appropriately translated, cross-culturally adapted, and validated.^53^

As PROM use for ACL injuries continues to grow, it is important to ensure these tools are utilized appropriately, particularly as outcomes after ACL surgery are more complex than graft retear rates. As such, the purpose of this study was to evaluate the adaptation quality of PROMs for ACL injuries in the Spanish language and to assess the psychometric properties of PROMs for ACL injuries in the Spanish language. We hypothesized that PROMs for ACL injuries in Spanish-speaking patients will exhibit inadequate adaptation quality and psychometric properties.

## Methods

### Literature Search

This review was conducted according to the PRISMA (Preferred Reporting Items for Systematic Reviews and Meta-Analyses) guidelines.^36^ We searched the PubMed, Cinahl via EBSCO, Medline via Ovid, Embase, and Web of Science databases on March 3, 2023, with the objective of identifying all published studies on linguistic and cultural adaptations and validation of instruments pertaining to ACL injuries in Spanish. We utilized the names of all of the PROMs that are described for ACL injuries, as guided by previous work.^31^ We considered studies that evaluated lower extremity conditions and other traumatic conditions that included patients with ACL injuries. The searches were run using common terms related to PROMs; Spanish language and Spanish-speaking countries; cross-cultural adaptation or validity; and lower extremity, knee, or ACL. The full search strategy is shown in the Supplemental Material (available separately).

### Eligibility Criteria

There was no time restriction for the included studies. Our inclusion criteria were (1) studies related to linguistic or cultural adaptation of PROMs assessing ACL injuries in the Spanish language; (2) studies reporting the process of linguistic or cross-cultural adaptation to Spanish; (3) studies reporting testing of linguistic or cross-cultural adaptations to Spanish; (4) studies with a full-text original article; (5) studies published in peer-reviewed journals; (6) studies written in either English or Spanish; (7) studies that included adults aged ≥18 years who had sustained ACL injuries; and (8) studies with evidence levels of 1 to 4. Excluded were comments, letters, editorial guidelines, conference reports, and reviews.

The retrieved articles were uploaded to Rayyan, an open-source online platform used for systematic reviews, and duplicates were subsequently eliminated.^35^ Afterward, 2 independent reviewers (J.S., E.G.L.) screened the titles and abstracts, and the full-text articles were further assessed based on predetermined inclusion and exclusion criteria. Additional relevant studies that were missed during the primary search were identified by examining the references. The reviewers convened to discuss the included articles, and any disagreements were resolved through consensus. If a consensus was unachievable, a third reviewer (L.M.S.) was available to facilitate an agreement.

**Figure 1.**
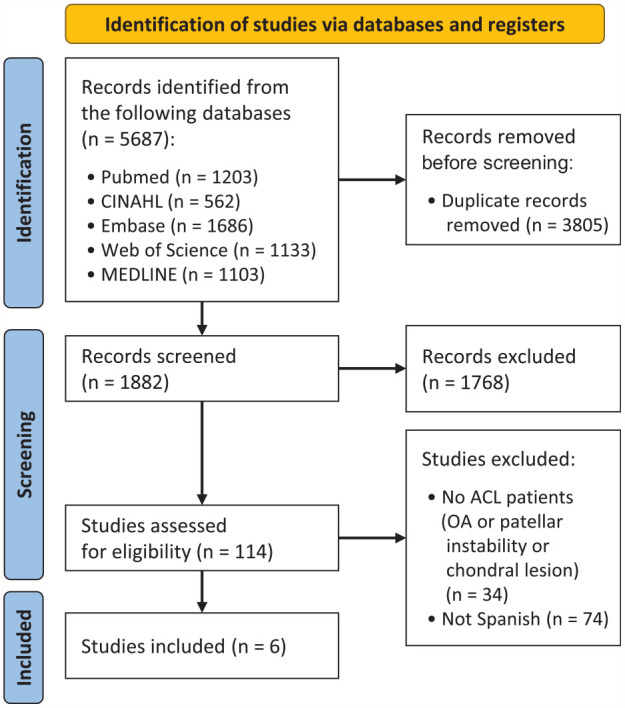
Flowchart summarizing the literature search, screening, and review. ACL, anterior cruciate ligament; OA, osteoarthritis.

The initial search yielded a total of 5687 studies. After removal of duplicates, 1882 studies remained for further analysis. Our team conducted a thorough review of the references, but no additional studies were identified. The literature search, screening, and review process is depicted in Figure 1.

### Data Extraction and Quality Assessment

The following information was extracted from each study: lead author, year of publication, inclusion criteria, sample size, age (mean and standard deviation), sex distribution, condition studied, country of study, and PROMs. The methodological quality of the included studies was evaluated by 2 reviewers (J.S., E.G.L.) using 3 checklists:

1. The Guidelines for the Process of Cross-Cultural Adaptation of Self-Reported Measures, which consists of 6 items that outline the standardized adaptation process for translating and culturally adapting an instrument.^4,15,54^
2. The Quality Criteria for Psychometric Properties of Health Status Questionnaires, which evaluates the content validity, criterion validity, construct validity, agreement, reliability, responsiveness, floor or ceiling effects, and interpretability of instruments.^49^
3. The Consensus-based Standards for the selection of health Measurement Instruments (COSMIN) Checklist for Cross-Cultural Validity, which evaluates the methodological quality of studies and the cross-cultural validity of a PROM.^33,34^ Cross-cultural validity measures the degree to which the performance of items on a translated or adapted instrument adequately reflects the performance of the original version.^33,34^ We utilized the “worst score counts” principle to evaluate the COSMIN checklist (ie, the lowest rating of any standard in the box is recorded).^24,46^

Agreement between the 2 reviewers was evaluated using the quadratic weighted κ statistic (κ_w_). If κ_w_ was >0.75, the results were adopted. Disagreements were resolved through consensus discussion or with the involvement of a third reviewer (L.M.S.).

### Level of Evidence Assessment

We determined the level of evidence for each PROM by combining the results of the studies for each domain. In accordance with previous investigation, we considered the number of studies, the methodological quality of the studies, and the consistency of the results.^7,24,32,40,49-51^ Sample size was evaluated in accordance to previous investigations and as dictated by COSMIN, in which sample sizes of >100 were considered excellent, 50 to 100 good, 30 to 50 fair, and <30 poor.^24,32,33,40,51^ We rated the measurement properties for each PROM as strong, moderate, limited, or conflicting evidence based on these factors.^23,31,39,50-52^

## Results

Ultimately, 6 studies were selected for final review,^2,8-10,39,43^ of which 4 were published in English^2,8,9,43^ and 2 in Spanish.^10,39^ The evaluated studies included 4 PROMs: the Lysholm knee score, the Anterior Cruciate Ligament–Return to Sport After Injury (ACL-RSI), the Lower Extremity Functional Scale (LEFS), and the Lower Limb Functional Index (LLFI). Three of the studies focused specifically on ACL injuries,^2,39,43^ and the other 3 studies examined a range of conditions including ACL injuries.^8-10^ The study populations comprised persons from 3 different countries; the key characteristics of the included studies are listed in Table 1.

**Table 1:** Characteristics of Included Studies^*a*^

Characteristic	Arroyo Morales (2019)^2^	Peña (2021)^39^	Sala-Barat (2019)^43^	Cruz-Díaz (2006)^8^	Dell’Era (2016)^10^	Cuesta-Vargas (2014)^9^
Inclusion criteria	Adult patients with ACL injury	Adult patients with ACL injury	Football players undergoing primary ACLR with or without associated meniscal injury	Various acute/subacute/chronic MSK diagnoses (9% were lower extremity ligamentous injuries)	Various MSK injuries (22% were lower extremity ligamentous injuries)	Various lower limb injuries (24% were ligamentous injuries of the knee)
Sample size	95	93	114	132	127	136
Age, y (mean ± SD)	21.8 ± 5.4	M: 36; F: 45^*b*^	21.8 ± 5.22	27.11 ± 6.22	43 ± 17.1	48 ± 19^*c*^
Sex, n (%)	M: 64 (67); F: 31 (33%)	M: 40 (43); F: 53 (57%)	M: 87 (85); F: 17 (15%)	M: 73 (55); F: 59 (45)	M: 59 (47); F: 68 (54%)	M: 62 (46); F: 74 (54%)
Country	Spain	Colombia	Spain	Spain	Argentina	Spain
PROM evaluated	Lysholm	Lysholm	ACL-RSI	LEFS	LEFS	LLFI

a ACL, anterior cruciate ligament; ACLR, anterior cruciate ligament reconstruction; ACL-RSI, Anterior Cruciate Ligament–Return to Sport After Injury; F, female; LEFS, lower extremity functional scale; LLFI, lower limb functional index; M, male; MSK, musculoskeletal.

b No SD reported.

c Age 50 ± 19 years for patients with knee injuries.

### Quality Assessment of the Adaptation Process

The interrater reliability for the quality assessment of the adaptation process yielded a κ_w_ value of 0.94, indicating substantial agreement. As shown in Table 2, all 6 studies included the initial translation, synthesis of the translation, and back-translation steps.^2,8-10,39,43^ Four studies incorporated an expert committee as well as testing of the prefinal version.^8,10,39,43^ Three studies included an appraisal of the adaptation process^10,39,43^; notably, these same 3 studies completed all 6 steps of the adaptation process.

**Table 2:** Quality Assessment of the Adaptation Process and Quality Criteria for Measurement Properties^*a*^

Assessment	Arroyo Morales (2019)^2^	Peña (2021)^39^	Sala-Barat (2019)^43^	Cruz-Díaz (2006)^8^	Dell’Era (2016)^10^	Cuesta-Vargas (2014)^9^
Quality assessment of the adaptation process						
Initial translation	+	+	+	+	+	+
Synthesis of the translations	+	+	+	+	+	+
Back-translation	+	+	+	+	+	+
Expert committee	o	+	+	+	+	o
Testing the prefinal version	+	+	+	-	+	-
Appraisal of the adaptation process	o	+	+	-	+	-
Quality criteria for measurement properties						
Criterion validity	+	o	o	o	o	+
Construct validity	+	o	+	+	?	+
Content validity	-	?	?	?	?	-
Internal consistency	+	+	+	+	+	+
Agreement	o	+	o	+	o	+
Reliability	+	+	+	+	+	+
Floor/ceiling effect	+	o	+	+	o	o
Responsiveness	-	o	o	-	+	-
Interpretability	o	o	o	+	?	o

a +, performed; ?, indeterminate rating; -, not performed, o, unavailable or not clear.

### Measurement Property Methodology

The κ_w_ value for interrater reliability regarding the assessment of measurement properties was 0.95, indicating substantial agreement (Table 2). All of the included studies were rated positively for internal consistency, assessed via Cronbach alpha, and for reliability, assessed via intraclass correlation coefficient (ICC). Two studies reported both construct and criterion validity,^2,9^ whereas no other study had information available on criterion validity. Four studies received an indeterminate rating for content validity,^8,10,39,43^ as they lacked a clear description of the measurement aim, target population, or concepts being measured. Meanwhile, 2 studies were rated negatively with regard to content validity,^2,9^ as they failed to describe the target population. None of the studies evaluated all of the measurement properties.

### COSMIN Cross-Cultural Validity

The κ_w_ value for interrater reliability regarding the cross-cultural validity assessment was 0.89, indicating substantial agreement. None of the studies demonstrated all 14 domains required for cross-cultural validity (Table 3). In addition, only 2 studies reported the percentage of missing items (item 1).^2,9^ Notably, all studies reported an adequate sample size (item 3), and in all studies, the items were translated forward and backward (item 7).

**Table 3:** COSMIN Checklist for Cross Cultural Validity^*a*^

Checklist Item^*b*^	Arroyo Morales (2019)^2^	Peña (2021)^39^	Sala-Barat (2019)^43^	Cruz-Díaz (2006)^8^	Dell’Era (2016)^10^	Cuesta-Vargas (2014)^9^
1	+	-	-	-	-	+
2	+	-	-	-	-	+
3	+	+	+	+	+	+
4	+	+	+	+	+	+
5	-	+	+	+	o	+
6	o	+	+	+	+	+
7	+	+	+	+	+	+
8	o	+	+	+	+	o
9	o	+	+	+	+	o
10	+	+	+	-	+	-
11	+	-	+	-	+	-
12	+	o	+	+	+	o
13	+	-	-	-	-	-
14	+	-	+	+	-	+

a +, yes; -, no; o, unavailable or not clear. COSMIN, Consensus-based Standards for the selection of health Measurement Instruments; HR-PRO, health-related patient-reported outcomes.

b Items: 1, Was the percentage of missing items given? 2, Was there a description of how missing items were handled? 3, Was the sample size included in the analysis adequate? 4, Were both the original language in which the HR-PRO instrument was developed, and the language in which the HR-PRO instrument was translated described? 5, Was the expertise of the people involved in the translation process adequately described? 6, Did the translators work independently from each other? 7, Were items translated forward and backward? 8, Was there an adequate description of how differences between the original and translated version were resolved? 9, Was the translation reviewed by a committee? 10, Was the HR-PRO instrument pretested to check interpretation, cultural relevance of the translation, and ease of comprehension? 11, Was the sample used in the pretest adequately described? 12, Were the samples similar for all characteristics except language and/or cultural background? 13, Were there any important flaws in the design or methods of the study? 14, For Classical Test Theory: was confirmatory factor analysis performed? For Item Response Theory: Was differential item function between language groups assessed?

### Level of Evidence Assessment

A κ_w_ value of 0.826 was achieved for interrater reliability regarding the level of evidence assessment, indicating substantial agreement between the reviewers. The ACL-RSI achieved the highest level of evidence, with 3 of the 9 domains demonstrating moderate evidence (Table 4). No domain of any PROM demonstrated strong evidence. Internal consistency and reliability showed the most evidence across all PROMs, while interpretability was available for only 1 study.^8^

**Table 4:** Ratings of Measurement Properties for Level of Evidence^*a* *b*^

Domain	Lysholm	ACL-RSI	LEFS	LLFI
Criterion validity	+	NA	NA	+
Construct validity	+	+	+	+
Content validity	+	+	+	NA
Internal consistency	++	++	++	+
Agreement	+	NA	+	+
Reliability	++	++	++	+
Responsiveness	+	++	+	NA
Floor and ceiling	+	+	+	NA
Interpretability	NA	NA	+	NA

a ACL-RSI, Anterior Cruciate Ligament–Return to Sport After Injury; LEFS, lower extremity functional scale; LLFI, lower limb functional index; NA, not available (not performed or described).

b Grading: +++ or −−−, multiple studies of good quality OR 1 study of excellent quality: strong evidence of positive/negative result; ++ or −−, multiple studies of fair quality OR 1 study of good quality: moderate evidence of positive/negative result; + or −, 1 study of fair quality: limited evidence of positive/negative result; +/−, conflicting findings; ?, only studies of poor quality: unknown, due to poor methodological quality.

## Discussion

In this review, we identified 4 instruments, none of which demonstrated appropriate adaptation or robust psychometric properties. Our findings highlight the need for improvement in outcome measurement for Spanish-speaking patients with ACL injuries and the potential for mismeasurement and inappropriate application of PROM results in this population. Given the growing use of PROMs in guiding care and assessing treatment of patients with ACL injuries, appropriate utilization across diverse patient groups is crucial. Linguistic and cultural adaptation of PROMs promotes accurate measurement, applicability, and patient engagement. This also leads to the inclusion of a more diverse patient population in orthopaedic research, particularly with the ability to capture language-based outcomes.

With increasing cross-national research, it is crucial to adapt PROMs to specific populations to ensure appropriate measurement. This is illustrated by the growing body of evidence demonstrating that a patient's language and culture can significantly influence their PROM scores.^1,6,38,53^ Notably, 3 studies (of 3 PROMs) followed all 6 steps of the comprehensive adaptation process outlined by Beaton et al.^3,10,39,43^ The 3 studies that did not include all steps of the adaptation process completed the initial steps but failed to test the prefinal version and appraise the adaptation process (Table 2).^2,8,9^ These data demonstrate superior cultural adaptation as compared with other conditions in orthopaedics; for example, a similar evaluation of PROMs for patients with distal radius fractures demonstrated that no studies followed and reported all 6 steps of cross-cultural adaptation.^25^ While the cross-cultural adaptation of PROMs evaluating ACL injuries in the Spanish-speaking population may be superior to that of other conditions, high-quality PROM adaptation can be enhanced by following standardized guidelines and documenting the process.^3^

Psychometric evaluation, typically conducted after the adaptation of a PROM, is important to understand and ensure the validity of a PROM in measuring the intended constructs. Construct validity, internal consistency, reliability, and floor/ceiling effect were reliably measured by all of the included studies.^2,8-10,39,43^ Notably, interpretability was only reported in 1 study.^8^ Given the variance of literacy levels of patients, it is important to have a PROM that is easily readable after being translated.^50^ Finally, the content validity of every study was either negatively rated or indeterminant. Content validity, the extent to which a PROM measures the concept of interest (eg, physical function) in a target population (eg, Spanish-speaking patients with ACL injuries) is a critical step in the evaluation and validation of a PROM and is considered to be the most important measurement property of a PROM, such that its importance is stressed by the US FDA and the European Medicines Agency.^7,12,51,52^ Notably, the lack of content validity negatively affects other measurement properties (eg, responsiveness, internal consistency), and may indicate a PROM is ineffective at measuring a specific concept in a specific population.^7,12,51,52^ Ensuring that components of the Quality Criteria for Psychometric Properties of Health Status Questionnaires guidelines are addressed and documented appropriately helps ensure the proper use of PROM tools and understand opportunities for improvement.^13,49^

Finally, the COSMIN checklist guides the assessment of cross-cultural validity, gauging how well translated or culturally adapted instruments capture the original version's item performance.^49^ Our research revealed that no PROMs specifically related to ACL injuries had undergone rigorous cross-cultural validation for use in Spanish-speaking populations. Sala-Barat et al^43^ was the most rigorous, reporting 11 out of 14 items. Similarly, it was not obvious whether multiple studies had included data pertaining to some of the items (Table 3). It is possible that the authors of these studies were not specifically following the COSMIN guidelines, which highlights the importance of utilizing a systematic approach to the cross-cultural adaptation of PROMs. Future research should detail how missing data were handled, address committee member disagreements, and describe the translation committee. These aspects are crucial for establishing PROM validity and reliability in diverse populations.

The lack of appropriately adapted and validated instruments has implications for both research and clinical practice. From a research perspective, multiple studies have demonstrated the lack of diversity of patients included in research studies, particularly randomized controlled trials. For example, a systematic review evaluating orthopaedic randomized controlled trials demonstrated that the reporting of race and ethnicity of study participants occurred in 7% and 3% of publications, respectively.^37^ This phenomenon has been demonstrated in the spine and hand literature as well.^11,22^ The lack of appropriately translated and adapted PROMs may contribute to the lack of diversity in trials and has been cited as a barrier to inclusion of diverse populations in research.^20,45^ Because clinical practice guidelines (ie, tools based upon best available evidence to guide treatment) are informed by these research studies, it is critical these studies include diverse patient populations such that the clinical practice guidelines are broadly applicable. Similarly, there is growing evidence that demonstrates differences in outcomes based on race, ethnicity, and language.^23,41,42^ As PROM use grows in guiding clinical care and evaluating outcomes, the appropriate adaptation and use of these tools is critical to prevent health care disparities.

### Limitations

This review has limitations. First, the scope of the review was confined solely to peer-reviewed articles, thus potentially introducing publication bias. This bias may have also affected the validity of our results, given that studies presenting positive outcomes are more likely to receive publication. Further, we recognize that many other PROMs exist that may have been translated and adapted for Spanish-speaking patients with ACL injuries. For example, many Patient Reported Outcomes Measurement Information System (PROMIS) tools have been translated and may be used in research and in clinical practice; however, our search strategy did not identify any relevant development, adaptation, or validation studies specific for this population. Second, the inclusion criteria only considered studies with complete original texts, which may have excluded relevant research solely available in abstract form or conference reports. Another limitation arose from the absence of a gold standard by which to compare with the newly created or adapted questionnaire (eg, in evaluating criterion validity). Identifying such a measure for ACL injuries would permit a more accurate evaluation of each questionnaire's reliability. Furthermore, we did not seek clarification or retrieval of unpublished data from the authors of the included papers.

## Conclusion

Our study demonstrated that, despite ACL injury prevalence and patient diversity, there was insufficient evidence supporting the adaptation quality and psychometric properties of PROMs for Spanish-speaking patients. We identified 4 instruments, none of which demonstrated appropriate adaptation or robust psychometric properties. The findings highlight the need for improvement in PROMs for Spanish-speaking patients and the potential for mismeasurement and inappropriate application of PROM results in this population. These improvements in the quality and psychometric properties may be possible with adherence to standard guidelines and reporting processes.
